# UV‐Induced Fluorescence in the Balance: Mate Choice and Predation Risk in the Female Ornamented Jumping Spiders

**DOI:** 10.1111/1749-4877.12979

**Published:** 2025-04-09

**Authors:** Yingna Zhou, Long Yu, Xiaoyan Wang, Daiqin Li, Xin Xu

**Affiliations:** ^1^ Centre for Behavioural Ecology and Evolution, School of Life Sciences Hubei University Wuhan Hubei China; ^2^ Department of Biological Sciences National University of Singapore Singapore; ^3^ College of Life Sciences Hunan Normal University Changsha Hunan China

**Keywords:** male mate choice, predation risk, trade‐offs, UV‐induced fluorescence, visual signaling

## Abstract

The adaptive significance of female ornamentation remains a central question in evolutionary biology, with ultraviolet (UV)‐induced fluorescence emerging as a key area of interest. This study investigates the potential adaptive advantages of female‐specific UV‐induced fluorescence in male mate choice and predation risk, as fitness costs, using two species of ornate jumping spiders *Phintella vittata* and *Ph. bifurcilinea*. In these species, the palps of adult females exhibit UV‐induced fluorescence, offering a compelling model to explore the interplay of sexual and natural selection acting on female ornamentation. In male mate‐choice trials, males were presented with a choice between a fluorescent (F+, UV‐visible) and a non‐fluorescent (F–, UV‐blocked) female. Males showed pronounced mate preference for F+ females over F– females, that is, spending significantly more time interacting with F+ females, suggesting that fluorescence serves as a sexually selected signal. To assess the potential costs of fluorescence, we tested its effect on predation risk using the spider‐eating jumping spider *Portia xishan* as a predator under F+ and F– conditions. Predation rates were significantly higher for F+ females than for F– females, indicating that UV‐induced fluorescence increases detectability by predators. These findings provide empirical evidence of a trade‐off: While fluorescence enhances male mate preference, it also increases predation risk. This study is the first to demonstrate the dual roles of fluorescence in sexual signaling and predation in female jumping spiders, challenging traditional male‐centric perspectives on mate choice. By integrating behavioral and ecological approaches, this work offers new insights into the evolutionary trade‐offs associated with female sexually selected traits.

## Graphical abstract

This study investigates the potential adaptive advantages of female‐specific UV‐induced fluorescence in male mate‐choice and predation risk, as fitness costs, using two species of ornate jumping spiders *Phintella vittata* and *Ph. bifurcilinea*. Our findings provide empirical evidence of a trade‐off: While fluorescence enhances male mate preference, it also increases predation risk.

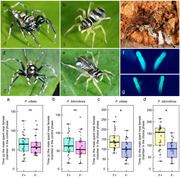

## 1 Introduction

The evolution of animal ornamentation, such as UV‐induced fluorescence, is often driven by the opposing forces of sexual and natural selection, creating a dynamic balance between traits that enhance reproductive success and those that increase survival costs (Andersson 1994; Crowell et al. 2024; Finkbeiner et al. 2017; Lerch and Servedio 2023). Sexual selection favors conspicuous traits, such as vibrant coloration, intricate displays, or elaborate structures, that increase an individual's attractiveness to mates or dominance over rivals, thereby improving reproductive success (Andersson 1994; Kyogoku and Sota 2021). However, natural selection imposes constraints on these traits, as their conspicuousness can make individuals more vulnerable to predation or reduce their energy efficiency (Lerch and Servedio 2023; Modarressie et al. 2013). This interplay creates an evolutionary trade‐off, where traits must optimize fitness benefits in mating success while minimizing survival costs.

Among the wide array of sexually selected traits, UV‐induced fluorescence has garnered increasing attention due to its intriguing optical properties and ecological roles. Fluorescence occurs when organisms absorb electromagnetic radiation at shorter wavelengths (e.g., UV; wavelengths <400 nm) and subsequently re‐emit it at longer, human‐visible wavelengths (Marshall and Johnsen 2017). This phenomenon spans a wide range of taxa, from single‐celled organisms and marine invertebrates to terrestrial arthropods and vertebrates (Marshall and Johnsen 2017). Fluorescence is typically mediated by specialized molecules, such as fluorescent proteins, pigments, or nanostructures, which facilitate the transformation of energy and its emission (Johnsen 2012; Lagorio et al. 2015; Salih et al. 2000). The occurrence of fluorescence is widespread, but its evolutionary significance remains poorly understood, particularly in the context of sexual and natural selection pressures.

The adaptive significance of fluorescence varies widely among taxa. In marine organisms such as jellyfish and corals, fluorescence often serves functions like photoprotection, prey attraction, or camouflage in blue‐light environments (Clarke et al. 2024; Lagorio et al. 2015). In contrast, terrestrial species more commonly use fluorescence in visual signaling, including mate attraction, species recognition, and predator avoidance (Andrews et al. 2007; Arnold et al. 2002; LeBas and Marshall 2000; Meadows et al. 2014; Whitcher 2024; Whitcher et al. 2024a). UV‐induced fluorescence is particularly significant in terrestrial animals, where it enhances signal visibility in UV‐rich environments under natural sunlight (Lim et al. 2007; Pearn et al. 2001; Whitcher et al. 2024b). Despite the prevalence of these traits, the evolutionary drivers behind UV‐induced fluorescence remain poorly understood. It is likely that fluorescence represents an evolutionary trade‐off, shaped by the competing pressures of sexual selection and natural selection. Sexual selection may favor fluorescence as an honest signal of health or genetic quality, while natural selection may impose survival costs, such as increased predation risk, particularly for predators with visually guided predators, such as birds, reptiles, and certain arthropods (Arnold et al. 2002; Camacho et al. 2019; Cronin et al. 2014; Czarnecki et al. 2022; Whitcher et al. 2024a). While this trade‐off is central to elucidating the adaptive significance of fluorescence, empirical tests are lacking (Czarnecki et al. 2022; Whitcher et al. 2024a).

Although sexual selection has traditionally focused on male ornamentation, with males often exhibiting more conspicuous traits in sexually dimorphic species, there is growing evidence that females, too, can evolve ornamentation through sexual selection (Chenoweth and McGuigan 2010; Rosenthal 2017; Schlupp 2021). In the case of the ornate jumping spider genus *Phintella* Strand, 1906, some species display female‐specific UV‐induced fluorescence in their palps, a trait rarely seen in males (D. Li, unpublished data). This pattern raises important questions about the role of sexual selection in shaping female ornamentation, suggesting that if males prefer F+ (UV‐visible) females, fluorescence may serve as a sexually selected trait. However, fluorescence may also impose survival costs, particularly by increasing predation risk (Arnold et al. 2002; Czarnecki et al. 2022; Whitcher et al. 2024a). Predators with optical sensitivity within the range of fluorescence, such as birds, reptiles, and spider‐eating jumping spiders, may more easily detect F+ individuals in UV‐rich environments (Cronin et al. 2014; Czarnecki et al. 2022). Thus, fluorescence may represent an evolutionary trade‐off, simultaneously enhancing mate attraction and increasing predation risk, with environmental factors such as habitat lighting influencing the balance of these effects.

The genus *Phintella* offers a unique opportunity to explore these dynamics, as it exhibits sexually dimorphic UV‐induced fluorescence: Females display prominently F+ palps, while males do not (D. Li, unpublished data). This pattern challenges conventional assumptions about sexual selection and provides a model to study the interplay between mate choice, predation risk, and environmental factors. Despite growing interest in UV‐induced fluorescence, several key questions remain unanswered. Most research has focused on male‐biased ornamentation, leaving female‐biased traits largely unexplored. Moreover, the potential trade‐offs associated with fluorescence, particularly regarding predation risk, are poorly understood. These gaps highlight the need for integrative studies that examine both the reproductive benefits and survival costs of fluorescence in female ornamentation.

This study addresses these gaps by investigating the adaptive significance of female fluorescence in two species of ornate jumping spiders, *Phintella vittata* (C. L. Koch, 1846) and *Ph. bifurcilinea* (Bösenberg & Strand, 1906). Specifically, we aim to: (1) determine whether UV‐induced fluorescence in female palps influences male mate choice and (2) assess whether fluorescence increases predation risk from UV‐sensitive predators. To test these hypotheses, we conducted mate‐choice trials in which males were presented with a choice between an F+ (UV‐visible) and an F– (UV‐blocked) female. We predict that males will prefer F+ females, indicating that fluorescence serves as a sexually selected signal. To evaluate the survival costs of fluorescence, we exposed F+ and F– females to the spider‐eating jumping spider *Portia xishan* Xu, Peng & Li, 2021 under controlled lighting conditions. We predict that F+ females will experience higher predation rates, reflecting the increased visibility of F+ signals to predators. By examining the trade‐offs between mate attraction and predation risk, this study provides novel insights into the evolutionary ecology of fluorescence, with broader implications for understanding how sexual and natural selection shape animal signals.

## 2 Materials and Methods

### 2.1 Collection and Maintenance of Spiders

**FIGURE 1:**
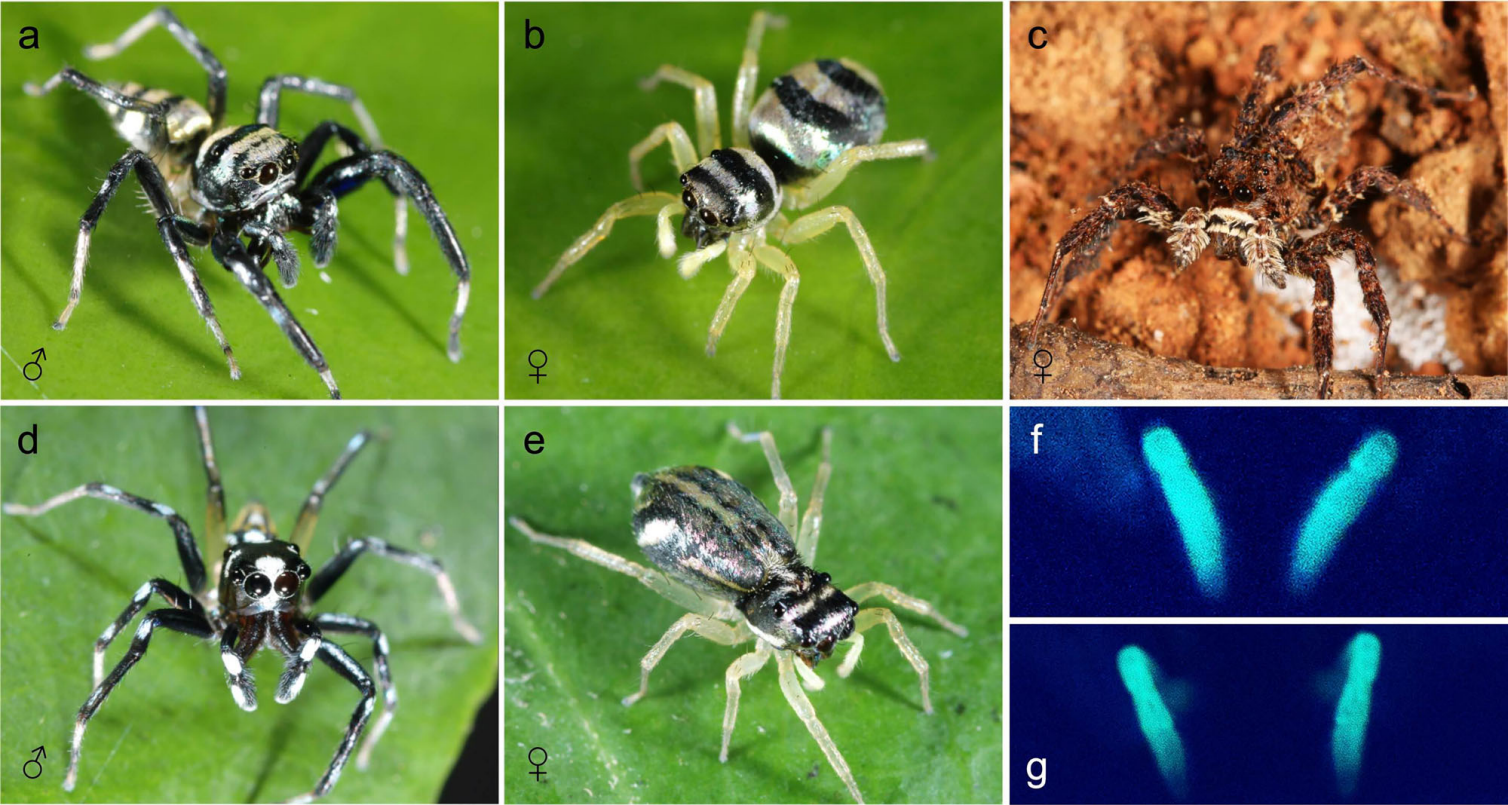
The color variation in study spiders: (a) Male *Phintella vittata*; (b) female *Ph. vittata*; (c) female *Portia xishan;* (d) male *Ph. bifurcilinea*; (e) female *Ph. bifurcilinea*; fluorescing palps of (f) *Ph. vittata* female and (g) *Ph. bifurcilinea* female under UV light (Photo: Daiqin Li and Long Yu).

The jumping spider genus *Phintella* is widely distributed across tropical Asia. We collected large juveniles and subadult individuals (one molt away from adulthood) of *Ph. vittata* and *Ph. bifurcilinea* from the Xishuangbanna Tropical Botanic Garden, Yunnan Province, China, and the Diaoluoshan National Forest Park, Hainan Province, China, in 2012 and 2013, as well as from Singapore in 2025. Spiders were reared to adulthood in the laboratory to ensure their virginity and monitor their post‐maturation age (age after reaching adulthood; tested within 20 days of maturation). Individuals of *Po. xishan* were collected from Western Hill, Kunming, Yunnan Province, China (Figure 1). Each spider was housed individually in cylindrical plastic containers (diameter × height: 3 × 6 cm) and provided with an *ad libitum* diet of fruit flies (*Drosophila melanogaster*) twice a week. Prior to testing, *Portia* individuals were starved for a week. All spiders were maintained under controlled environment conditions (RH: 80%–90%; temperature: 25 ± 1°C; light cycle: 12‐h light: 12‐h dark, lights on at 8 a.m.). Maintenance protocols followed established guidelines for salticids (Li et al. 2008a, 2008b; Lim and Li 2004, 2006).

### 2.2 Qualitative Assessment of UV‐Induced Fluorescence in Female Palps

To document the UV‐induced fluorescence of female palps, we placed an anesthetized *Phintella* individual on a black‐matt surface to provide a contrasting background. Using a fresh specimen preserved the integrity of the fluorescence signals. Illumination was provided by a dark tube (Hitachi BL/B), which selectively excited the green fluorescent pigments in the adult female's palps. Images were captured using a Nikon D800 digital SLR camera (Tokyo, Japan). White light served as the primary illumination source, facilitating clear visualization of both the specimen's morphology and fluorescence. To document the bioluminescent features, we followed the imaging protocol outlined by Lim et al. (2007) and Sparks et al. (2014). This included optimizing exposure settings and calibrating the light source to enhance the detection of UV‐induced fluorescence.

### 2.3 General Experimental Procedures

We conducted two experiments to examine the effects of UV‐induced fluorescence on mate choice (**Experiment 1**) and predation risk (**Experiment 2**) in both *Ph. vittata* and *Ph. bifurcilinea*. Both experiments were conducted during August 2012 and June 2013. Custom glass apparatuses were used in experiments optimized for video recording of mate choices (**Experiment 1**) and prey choices (**Experiment 2**) under full‐spectrum light transmission (300–700 nm). A dichotomous choice design was designed, with trials conducted under two contrasting lighting conditions: F+ (UV‐visible light, no filter) and F– (UV‐blocked light, filter present, Photonitech Pte. Ltd., Singapore) (Lim et al. 2007). A UV‐blocking filter was placed horizontally above the chamber to test whether responses varied with lighting conditions. Only virgin adult spiders with a known post‐maturation age (within 20 days of maturation) were used. To minimize potential effects from previous experiences, reused spiders were allowed to rest for at least 3 days after each trial.

Trials were recorded using two high‐definition digital video cameras (SONY HDR‐PJ600) positioned in front of the setup. Illumination was provided by 10 full‐spectrum Voltrarc Light tubes (110 W each, powered by a 120 V 50/60 Hz electronic ballast; SUPER‐TEK, Natural lighting.com, Houston, TX) and three UV‐emitting dark tubes (powered by a 230 V 50/60 Hz electronic ballast). The light sources were suspended 150 cm above the experimental setup, which was enclosed by a black screen to minimize external visual interference.

#### 2.3.1 Experiment 1: UV‐Induced Fluorescence–Dependent Mate Choice

**FIGURE 2:**
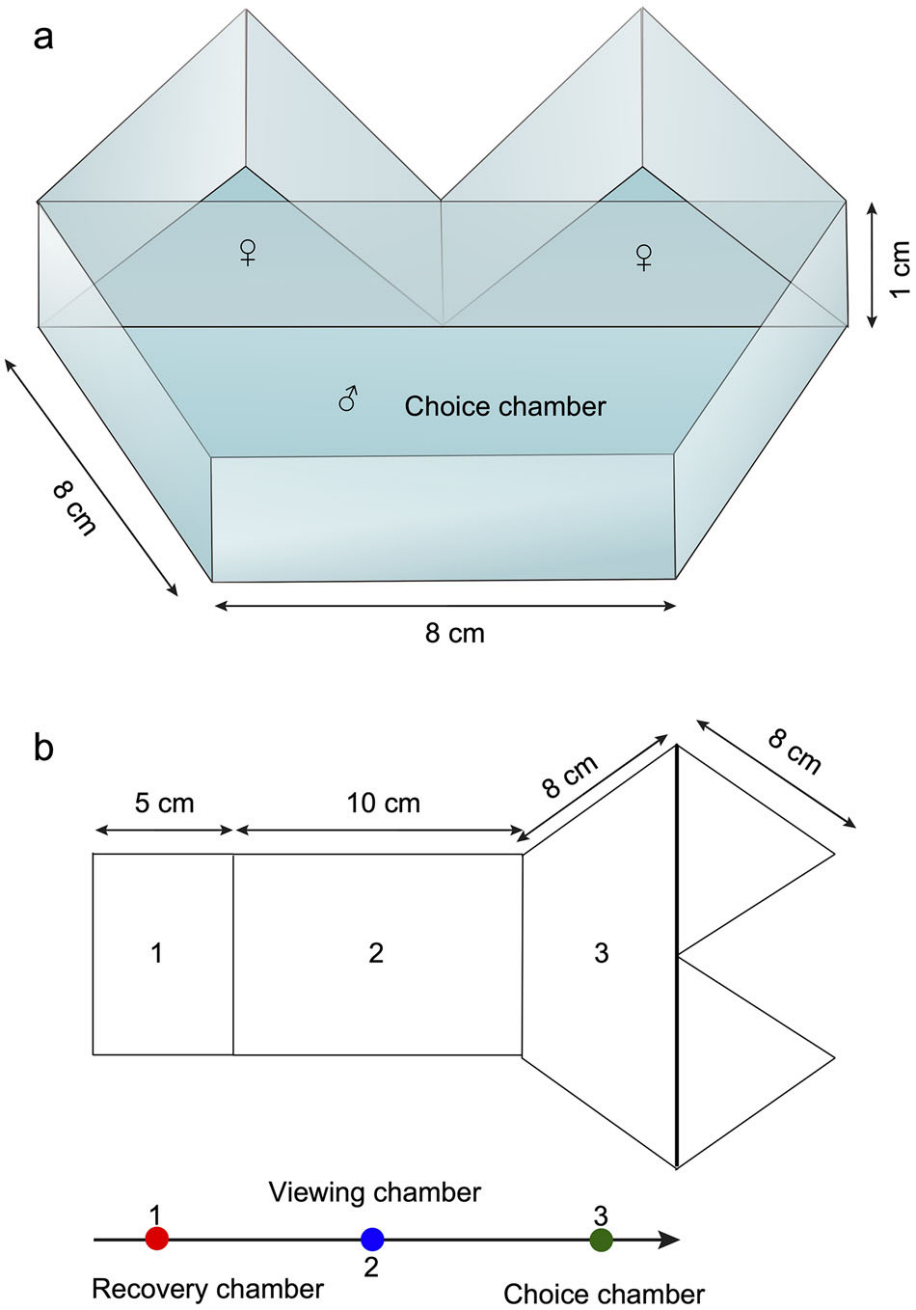
Experimental setups for male mate‐choice and prey‐choice trials: (a) Diagram of the mate‐choice apparatus used in Experiment 1. The choice chamber is used for male *Phintella* to choose mate, with two triangular‐shaped chambers holding the females, and (b) a diagram of the prey‐choice apparatus used in Experiment 2. Panel 1 shows the recovery chamber, where *Portia xishan* acclimates. Panel 2 shows the viewing chamber, where *Po. xishan* observes two *Phintella*. Panel 3 represents the choice chamber, where *Po. xishan* makes its prey choice between two *Phintella*. The two triangular‐shaped chambers house the *Phintella* females or a *Ph. vittata* female and a *Ph. vittata* male. The rightward arrow indicates the direction of *Portia*’s movement.

We conducted male mate‐choice trials in a three‐chambered apparatus comprising a central choice chamber (L × L × L × H = 8 × 8 × 16 × 1 cm) and two separate female chambers (L × L × L × H = 8 × 8 × 8 × 1 cm) (Figure 2a). Each trial consisted of a control phase (i.e., the female chambers were empty) and a choice phase. In the control phase, a male spider was placed in the central choice chamber and allowed to acclimate for 5 min. An opaque black cardboard barrier was placed between the choice and female chambers to prevent visual contact. After acclimation, the barrier was removed, and the male's behavior was recorded for 10 min to assess whether chamber preference was influenced by lighting conditions alone. The control phase ended when the barrier was replaced between the chambers.

At the beginning of the choice phase, two size‐matched females (*Ph. vittata*: mean ± SE carapace width (CW) = 1.45 ± 0.16 mm, *N* = 37; *Ph. bifurcilinea*: mean ± SE CW = 1.09 ± 0.19 mm, *N* = 34) were randomly placed in the female chambers. The choice phase began upon the removal of the barrier, allowing the male to visually access both females. Unlike some salticids that perform elaborate courtship displays (Jackson and Pollard 1997), *Phintella* females do not engage in distinct courtship displays to signal mate choice. Instead, salticid courtship often involves prolonged interactions where the male must sustain the female's attention long enough to secure mating (Jackson and Pollard 1997). During the 10‐min choice phase, male courtship behavior and female responses were recorded. Only adult, virgin males (21 *Ph. vittata* and 19 *Ph. bifurcilinea*) and females (37 *Ph. vittata* and 34 *Ph. bifurcilinea*) were used in the study. When males were used multiple times, different females were paired in each trial to avoid pseudo‐replication. A total of 22 male mate‐choice trials were conducted for *Ph. vittata* and 21 for *Ph. bifurcilinea*.

Three measures of male mate preferences were recorded for each trial: (1) time spent near the female chamber, defined as the time the male spent in proximity to each female's chamber during both the control and choice phases. This measure reflects the male's initial attraction, with longer time spent near a female indicating higher interest in a potential mate; (2) courtship duration, defined as the cumulative time the male spent performing courtship displays facing each female during the choice phase. This included “looking time,” defined as the time the male spent facing the direction of a female's chamber and engaging in face‐to‐face displays. Courtship duration is a well‐established indicator of male mating effort, where longer courtship reflects stronger investment influenced by the perceived quality of the female; and (3) female attention time, defined as the time each female spent looking at the male, reflecting her level of engagement. This measure is a crucial factor in mate‐choice decisions (Li et al. 2008b; Lim et al. 2008). Additionally, female body size (measured as CW) was recorded to account for potential size‐based mate preferences. In many species, larger individuals are often preferred due to their greater reproductive potential or overall fitness.

#### 2.3.2 Experiment 2: Survival Costs of UV‐Induced Fluorescence

The experiment aimed to test whether UV‐induced fluorescence influenced predator behavior by varying lighting conditions (F+ and F–) in prey chambers. Using *Po. xishan* as predators and *Phintella* females as prey, we conducted prey‐choice trails in a five‐chambered apparatus comprising a recovery chamber (L × B × H = 8 × 5 × 1 cm), a viewing chamber (L × B × H = 8 × 10 × 1 cm), a choice chamber (L × L × L × H = 8 × 8 × 16 × 1 cm), and two individual *Phintella* chambers (L × L × L × H = 8 × 8 × 8 × 1 cm) (Figure 2b). The recovery chamber allowed *Portia* to acclimate, while the viewing chamber ensured clear visibility of both *Phintella* female individuals, regardless of their orientation. In the choice chamber, *Portia* selected between two size‐matched *Phintella* individuals housed in separate triangular chambers.

The experiment consisted of a control phase followed by a choice phase. The control phase was conducted without *Phintella* in the chambers to rule out any bias in *Portia’*s choice based solely on lighting conditions. During this phase, *Portia* was acclimated in the recovery chamber for 5 min, separating from the viewing chamber by an opaque barrier. Following acclimation, the barrier was removed, and *Portia*’s behaviors were recorded as it moved through the apparatus (Figure 2b). A successful choice was defined as *Portia* touching the edge of a chamber, whether under the F+ or F– condition. If no choice was made within 30 min, the trial was abandoned and repeated the following day.

In the choice phase, two size‐matched *Phintella* females (*Ph. vittata*: mean ± SE CW = 1.41 ± 0.10 mm, *N* = 41; *Ph. bifurcilinea*: mean ± SE CW = 1.11 ± 0.13 mm, *N* = 36) were introduced into the chambers under F+ and F– conditions. Once the barrier was removed, *Portia* could visually access *Phintella* individuals, and its choices and behaviors were recorded. Only adult, virgin individuals were used: 41 *Ph. vittata* females, 36 *Ph. bifurcilinea* females, and 29 *Po. xishan* individuals. When *Po. xishan* was used multiple times, different females were paired in each trial to prevent pseudo‐replication. A total of 28 prey‐choice trials were conducted for *Ph. vittata* and 28 for *Ph. bifurcilinea*.

During both phases, we recorded the following: (1) time to traverse the choice chamber, defined as the time taken by *Portia* moving from the viewing chamber to the end of the choice chamber. This metric provides insight into *Portia*’s exploration and decision‐making process, indicating how quickly it assesses the environment and makes a choice; (2) first choice, defined as the first *Phintella* individual approached by *Portia*. This measure is critical for understanding how lighting conditions, such as UV‐induced fluorescence, influence predatory behavior. By tracking *Portia*’s first approach, we could assess whether certain visual cues or preferences drive its predatory actions; and (3) female movement time, defined as the time each *Phintella* female spent moving while *Portia* traversed the choice chamber. This measure accounts for the influence of prey activity on *Portia*’s decision‐making.

CW was also measured for *Phintella* and *Portia* individuals to test for potential size‐biased effects on predation. Prey size is an important factor in predator‐prey interactions, as larger individuals may be more challenging or less desirable for certain predators.

### 2.4 Data Analyses

We checked for the normality of all behavioral data using the Shapiro‐Wilk test before proceeding with statistical analyses. Parametric tests were applied to datasets that met the normality assumptions, while nonparametric tests were used for those that did not.

For data obtained from Experiment 1, we used a paired *t*‐test to compare the time males spent near the female chambers during the control phase. To assess whether males discriminated between females under different lighting conditions and made mate choices specific to each *Phintella* species during the choice phase, we used linear regression models. We first fitted a baseline model with only an intercept. We included the difference in time spent near females as the response variable. Female attention time difference, courtship duration difference, and female CW difference between F+ and F– females were used as continuous predictors, along with interaction terms among these predictors. This resulted in 16 candidate models for each species, including a null model (detailed in Table S1). Predictor collinearity was assessed using variance inflation factors (VIFs) through the *vif* function in the R package *car* (Fox and Weisberg 2018). Models with VIF values exceeding 2 were excluded. For *Ph. vittata*, m11 and m15 (Table S1) were excluded due to VIF values greater than 2. In contrast, all 16 models for *Ph. bifurcilinea* had VIF values below 2, indicating no collinearity issues. Models were compared using the corrected Akaike information criterion (AICc) for small sample sizes (Burnham and Anderson 2004) using the *model.sel* function in the R package *MuMIn* (Barton 2009). The best‐fitting model was identified as the one with the smallest AICc value, provided the ΔAICc compared to the second‐best model exceeded 2. When ΔAICc was less than 2, model averaging was conducted using the *model.avg* function in *MuMIn* to account for uncertainty in model selection.

For data from Experiment 2, chi‐square tests were used to compare *Portia*’s first choice under F+ and F– lighting conditions during both the control and choice phases. To evaluate the effects of *Phintella* female movement time and the relative CW of prey and predators on predation by *Po. xishan*, we performed binomial generalized linear models (GLMs). The binary choice of *Po. xishan* for paired females (F+  =  1; F– =  0) was included as the response variable, where only one female was ultimately preyed upon. *Phintella* female movement time difference, prey–predator CW ratio (calculated as the difference in CW between the two *Phintella* females divided by *Po. xishan* CW), and their interaction term between these two predictors were included as predictors. Six candidate models were proposed for each *Phintella* species, including a null model (Table S2). VIFs were assessed using the *vif* function. For *Ph. vittata*, all models had VIF values below 2. For *Ph. bifurcilinea*, model m5 was excluded due to a high VIF value. Model election followed the same AICc‐based criteria as in Experiment 1, ensuring robust statistical analysis. This approach accounted for the effects of multiple predictors and potential interactions while minimizing the risk of overfitting.

All data are reported as means ± SE. Statistical analyses were performed using R (version 4.4.0).

### 2.5 Ethical Note

The experiments comply with the ASAB/ABS Guidelines for the Use of Animals in Research and the current legal requirements of China, where the research was conducted. Spiders are not protected under animal protection laws in China. A minimum number of spiders required for each experiment were hand‐collected and housed in laboratory conditions designed to closely replicate their natural environment, as described above. At the end of the study, surviving spiders were released back to their original collection sites.

## 3 Results

### 3.1 Experiment 1: UV‐Induced Fluorescence–Dependent Mate Choice

**FIGURE 3:**
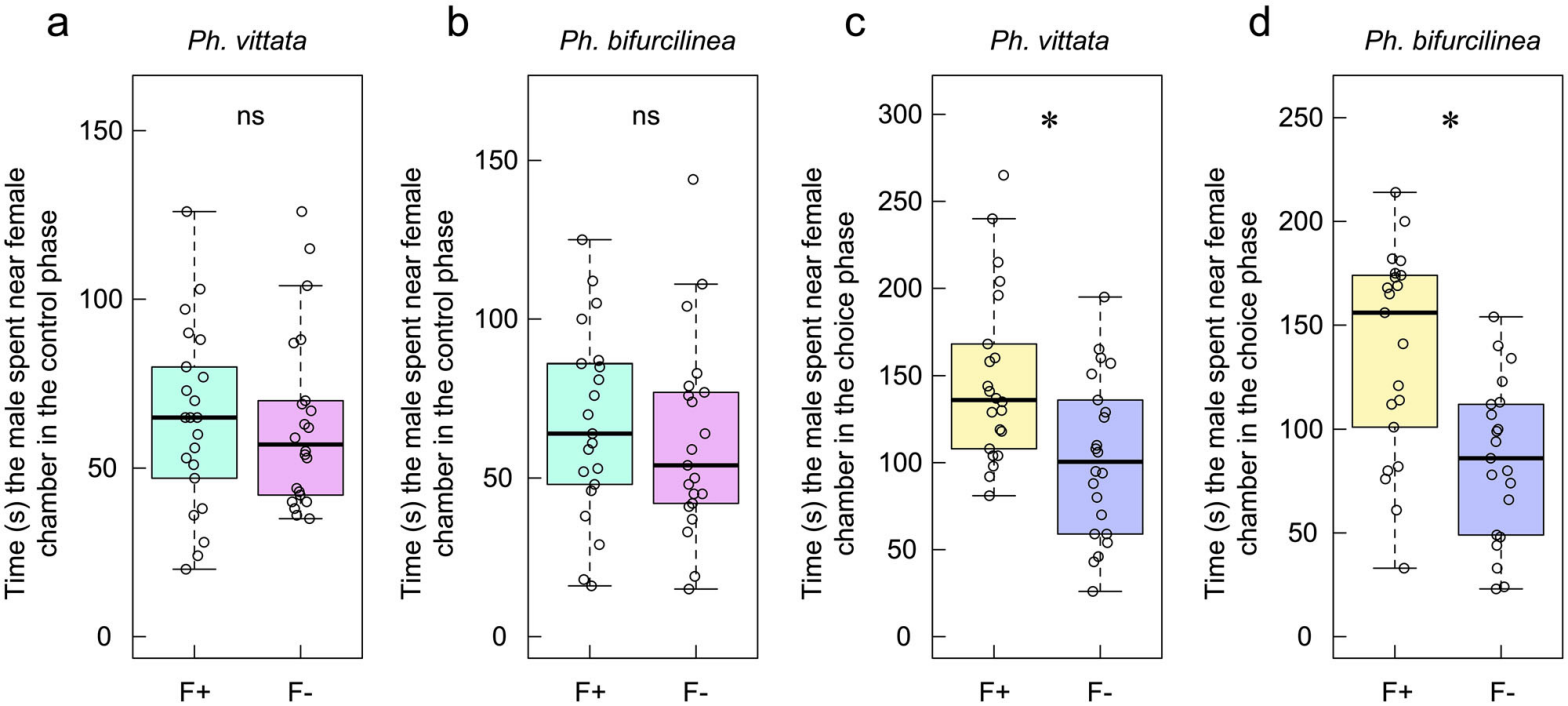
Results from male mate‐choice trials (Experiment 1) during the control and the choice phases. Boxplots illustrate the mate preferences of male *Phintella vittata* and *Ph. bifurcilinea* for F+ and F– female chambers under two lighting conditions: (a,b) time spent by *Ph. vittata* and *Ph. bifurcilinea* males near F+ and F– female chambers during the control phase, respectively; (c,d) time spent by *Ph. vittata* and *Ph. bifurcilinea* males near F+ and F– female chambers during the choice phase, respectively. Boxplots represent the median (central line), first and third quartiles (box), and minimum and maximum values (whiskers). ns: no significant difference. The asterisk above the bars indicates significant differences between F+ and F– lighting conditions.

During the control phase, males showed no significant preference between the F+ and F– observation chambers, spending a similar amount of time in each chamber for both *Phintella* species (*Ph. vittata*: *t*_22_ = 0.61, *p* = 0.549; *Ph. bifurcilinea*: *t*_21_ = 0.15, *p* = 0.880) (Figure 3a,b). However, in the choice phase, males of both species showed a significant preference for females with UV‐induced fluorescence (*Ph. vittata*: *β* = 1.10, adjusted SE = 0.15, *p* < 0.001; *Ph. bifurcilinea*: *β* = 0.80, adjusted SE = 0.26, *p =* 0.003, based on the conditional averaged model) (Table 1; Figure 3c,d). These results suggest that UV‐induced fluorescence plays an important role in male mate choice. Further analysis using the conditional averaged model indicated that male preference was not significantly influenced by female CW (*Ph. bifurcilinea*: *β* = −87.85, adjusted SE = 77.51, *z* = 1.05, *p* = 0.292), female attention time (*Ph. bifurcilinea*: *β* = 0.17, adjusted SE = 0.10, *z* = 1.66, *p* = 0.098), or the interaction between female attention time and courting duration (*Ph. bifurcilinea*: *β* = −0.002, adjusted SE = 0.001, *z* = 1.07, *p* = 0.285) (Table 1; Table S1). These findings suggest that UV‐induced fluorescence serves as a prioritized visual signal in male mate choice, independent of female body size or behavioral cues.

**TABLE 1:** Experiment 1: The top models (delta AICc < 2) with or without model average showing the effects of explanatory variables (predictors) on male mate preference of *Phintella vittata* (*n* = 22) and *Ph. bifurcilinea* (*n* = 21).

Model	Predictor	*β*	SE	*Z*	*p*
*Ph. vittata*					
	Intercept	10.80	10.80	1.00	0.329
	Courtship duration difference	1.10	0.15	7.27	<0.001^***^
*Ph. bifurcilinea*					
Full average	Intercept	34.51	12.85	2.52	0.012^*^
	Female attention time difference	0.11	0.11	0.94	0.349
	Courtship duration difference	0.80	0.26	2.93	0.003^**^
	Female attention time difference : Courtship duration difference	−0.0003	0.001	0.28	0.777
	Female CW difference	−0.12	0.41	0.28	0.782
Conditional average	Intercept	34.51	12.85	2.52	0.012^*^
	Female attention time difference	0.17	0.10	1.66	0.098
	Courtship duration difference	0.80	0.26	2.93	0.003^**^
	Female attention time difference : Courtship duration difference	−0.002	0.002	1.07	0.285
	Female CW difference	−87.85	77.51	1.05	0.292

*Note*: *n* represents the number of trials. Courtship duration difference is calculated as the courtship duration in the F+ condition minus the courtship duration in the F– condition. Female CW difference is calculated as the CW of females in the F+ condition minus the CW of females in the F– condition. Female attention time difference is calculated as the time spent by females looking at the male in the F+ condition minus the time spent in the F– condition.

Abbreviation: CW: carapace width.

*: *p* < 0.05, **: *p* < 0.01, ***: *p* < 0.001.

### 3.2 Experiment 2: Survival Costs of UV‐Induced Fluorescence

Chi‐square tests during the control phase indicated no significant preference by *Po. xishan* for either the F+ or F– sides when testing with *Ph. vittata* (*χ* ^2^ = 0.57, *p* = 0.450) and *Ph. bifurcilinea* (*χ* ^2^ = 1.29, *p* = 0.257). In the choice phase, fluorescence significantly influenced *Portia*’s prey preference, as *Po. xishan* consistently targeted F+ individuals when given a choice between an F+ or an F– female (*Ph. vittata*: χ^2^ = 9.14, *p* = 0.002; *Ph. bifurcilinea*: χ^2^ = 7.00, *p* = 0.008). Neither *Phintella* female movement time, predator–prey CW ratio alone, nor their interaction term had significant effects on *Portia*’s prey choice (Table 2; Table S2). These results suggest that fluorescence is a key factor in *Portia*’s prey choices, independent of the prey size, predator size, or *Phintella* behavior.

**TABLE 2:** Experiment 2: The top models (delta AICc < 2) with or without model average showing the effects of explanatory variables on predator preference of *Phintella vittata* (*n* = 28) and *Ph. bifurcilinea* (*n* = 28).

Model	Predictor	*β*	SE	*z*	*P*
*Ph. vittata*					
Full average	Intercept	1.40	0.50	2.68	0.007^**^
	Female movement time: Prey–predator CW ratio	0.27	0.32	0.81	0.420
Conditional average	Intercept	1.40	0.50	2.68	0.007^**^
	Female movement time : Prey–predator CW ratio	0.40	0.32	1.17	0.241
*Ph. bifurcilinea*					
	Intercept	1.70	0.60	2.85	0.004^**^
	Female movement time : Prey–predator CW ratio	0.39	0.20	1.89	0.059

*Note*: *n* represents the number of trials. Prey–predator CW ratio is calculated as the ratio of the difference in CW between two *Phintella* females to *Portia xishan* CW. **: *p* < 0.01.

Abbreviation: CW: carapace width.

## 4 Discussion

This study investigates the adaptive significance of UV‐induced fluorescence in female *Phintella* jumping spiders, exploring both its role in male mate choice and its potential survival costs in the form of increased predation risk. Our findings provide compelling evidence that UV‐induced fluorescence in the palps of *Phintella* females is a sexually selected trait, while also revealing potential evolutionary trade‐offs, including increased vulnerability to visually guided predators. These results highlight the complex interplay between sexual and natural selection in shaping the evolution of UV‐induced fluorescence as a signaling mechanism in *Phintella* jumping spiders.

### 4.1 UV‐Induced Fluorescence Influences Sexual Selection

Fluorescence expression in spiders varies widely across families and is often evolutionarily unstable (Andrews et al. 2007). Despite this instability, when present, fluorescence has been shown to influence mate preference (Andrews et al. 2007; Li et al. 2008b; Lim et al. 2007). Our first experiment aimed to explore whether UV‐induced fluorescence influences male mate choice in *Ph. vittata* and *Ph. bifurcilinea*. The results provide strong evidence that male spiders have a pronounced preference for females with UV‐induced fluorescence (F+), independent of body size or the amount of time females spent looking at males. This finding supports the hypothesis that fluorescence serves as an important visual signal in male mate choice, corroborating broader research on other species where conspicuous traits such as coloration, ornamentation, or fluorescence signal individual quality or fitness (Arnold et al. 2002; Camacho et al. 2019). In this context, fluorescence may act as an honest signal of female fitness, with males selecting mates that exhibit this trait due to its potential correlation with genetic quality or reproductive success. Given the high visibility of fluorescence under UV‐rich lighting conditions, this trait likely serves as a critical visual cue for males seeking to maximize reproductive success.

Interestingly, our results diverge from studies suggesting that body size often influences male preferences in many systems (Andersson 1994; Kyogoku and Sota 2021). Instead, fluorescence appears to be a specific, dominant criterion in male mate choice in *Phintella*. This aligns with findings from other salticids that prioritize visual cues over other traits (Li et al. 2008b; Lim et al. 2008). The absence of a size‐based preference in our study highlights the distinctiveness of fluorescence as an evolutionary driver in this context.

Our findings also prompt several questions. For example, while fluorescence is visually prominent, its interaction with other sensory modalities, such as chemical signals or movement, remains unclear. Second, our study was restricted to only two *Phintella* species due to collection constraints. Expanding the research to include additional species would help determine whether UV‐induced fluorescence influences mate choice more broadly across *Phintella* and other salticid species. Furthermore, the role of UV reflectance, which was simultaneously blocked in our UV filter experiments, cannot be entirely ruled out. Prior research shows that in *Phintella* species, including *Ph. vittata*, both males and females reflect UV light, which might also contribute to male preference (Li et al. 2008a). Further studies should disentangle the roles of UV‐induced fluorescence and UV reflectance in mate‐choice decisions to provide a more nuanced understanding of these visual signals.

### 4.2 UV‐Induced Fluorescence as a Cost of Predation Risk

While fluorescence clearly enhances male attraction to females, the second part of our study examined the potential survival costs associated with this trait. Using a well‐known spider‐eating predator *Po. xishan*, we demonstrated that fluorescence significantly increases predation risk. This is consistent with findings in other species where conspicuous traits, particularly in UV‐rich environments, elevate vulnerability to visually guided predators (Harland et al. 2012). Our results showed that *Po. xishan* consistently targeted F+ *Phintella* individuals, regardless of prey size or movement patterns, confirming that fluorescence itself, rather than brightness or other factors, increases *Phintella* spiders’ visibility to vision‐guided predators, driving predation risk.

These findings align with studies documenting the trade‐offs of conspicuous traits in other taxa (Lerch and Servedio 2023; Modarressie et al. 2013). For example, vibrant coloration in frogs is often linked to increased predation risk despite its role in sexual selection (Bell and Zamudio 2012). Similarly, UV‐induced fluorescence in *Phintella* represents a double‐edged sword: enhancing reproductive success while increasing predation risk. This dynamic suggests that the evolution of fluorescence may be constrained by ecological pressures, driving protective adaptations or limiting its expression in predator‐dense habitats.

### 4.3 Evolutionary Trade‐Offs and the Significance of Fluorescence

Our study supports the hypothesis that UV‐induced fluorescence in female *Phintella* is a sexually selected trait with dual consequences: enhancing male attraction while imposing predation risk. This trade‐off mirrors broader patterns observed in other species, where conspicuous traits advantageous for mate attraction are counterbalanced by survival costs (Modarressie et al. 2013; Whitcher et al. 2024a, 2024b). Such dynamics highlight the importance of considering both sexual and natural selection in understanding the evolution of conspicuous traits.

Moreover, this challenges traditional perspectives of sexual selection, which have predominantly focused on male ornamentation and courtship behaviors as the primary drivers of mate choice. By demonstrating that females can also evolve conspicuous traits subject to sexual selection, our study contributes to a growing body of literature highlighting the importance of female ornamentation and the complex dynamics of mate choice (Chenoweth and McGuigan 2010; Rosenthal 2017).

Furthermore, our study adds to the growing body of research on UV‐induced fluorescence, a phenomenon that has been widely studied in marine and aquatic systems but remains underexplored in terrestrial environments. While fluorescence in marine species often serves functions such as prey attraction or camouflage, in terrestrial species, it is often linked to visual signaling, particularly in the context of mate choice (Lagorio et al. 2015; Meadows et al. 2014; Whitcher 2024). This extends the current understanding of how fluorescence functions across diverse ecological contexts, offering a foundation for future research on terrestrial fluorescence.

Finally, environmental changes, such as UV radiation alterations and habitat disturbance, may further influence the evolution of fluorescence. Understanding how these factors interact with predation pressures will be crucial for predicting the evolutionary trajectories of the species relying on UV‐induced traits. Broader studies across habitats and species will help clarify how these trade‐offs operate in natural settings and inform conservation strategies for ecosystems affected by anthropogenic changes.

## 5 Conclusion

Our findings provide novel insights into the role of UV‐induced fluorescence in female *Phintella* jumping spiders. We demonstrate that fluorescence serves as a sexually selected signal that enhances male mate choice, likely increasing reproductive success. However, this conspicuous trait also imposes survival costs by increasing predation risk from visually guided predators. These findings highlight the intricate interplay between sexual and natural selection in shaping animal signals and underscore the need for integrative approaches to understanding the evolution of ornamental traits. Further research should expand on these findings to explore the ecological and evolutionary dynamics of fluorescence, including its interactions with habitat variation, predator–prey dynamics, and adaptive strategies to mitigate the costs of conspicuous traits.

## Ethics Statement

The experiments comply with the ASAB/ABS Guidelines for the Use of Animals in Research and the current legal requirements of China, where the research was conducted. Spiders are not protected under animal protection laws in China. A minimum number of spiders required for each experiment were hand‐collected and housed in laboratory conditions designed to closely replicate their natural environment, as described above. At the end of the study, surviving spiders were released back to their original collection sites.

## Conflicts of Interest

The authors declare no conflicts of interest.

## Supporting information




**Table S1** Experiment 1: Comparisons of 16 linear models to predict male mate‐choice.
**Table S2** Experiment 2: Comparisons of six generalized linear models to predict prey‐choice by *Po. xishan*

## Data Availability

Data and codes are available from Zenodo.
