# Supplementary material for: UV‐Induced Fluorescence in the Balance: Mate Choice and Predation Risk in the Female Ornamented Jumping Spiders
Source: Integr Zool. 2025 Apr 9;21(2):411–20. doi: 10.1111/1749-4877.12979 (PMC12971619; doi:10.1111/1749-4877.12979)
Supplement: Supplementary file 1 — Table S1 Experiment 1: Comparisons of 16 linear models to predict male mate‐choice. Table S2 Experiment 2: Comparisons of six generalized linear models to predict prey‐choice by Po. xishan [file INZ2-21-411-s001.docx]

**SUPPLEMENTARY MATERIALS**

**Tables**

**Table S1** Experiment 1: Comparisons of 16 linear models to predict male mate-choice

**Table S2** Experiment 2: Comparisons of six generalized linear models to predict prey-choice by *Po. xishan*

**Table S1** Experiment 1: Comparisons of 16 linear models to predict male mate-choice

| **Model** | **Predictor** | **df** | **logLik** | **AICc** | **Delta** | **Weight** | **VIF** |
| --- | --- | --- | --- | --- | --- | --- | --- |
|  | ***Ph. vittata*** | | | | | |  |
| **m6** | **Courtship duration difference** | 3 | -114.22 | 235.8 | 0.00 | 0.515 |  |
| **m2** | **Female CW difference + Courtship duration difference** | 4 | -113.73 | 237.8 | 2.05 | 0.185 |  |
| **m3** | **Female attention time difference + Courtship duration difference** | 4 | -114.19 | 238.7 | 2.97 | 0.116 |  |
| **m14** | **Courtship duration difference +** | 4 | -114.21 | 238.8 | 3.00 | 0.115 | 1.074 |
|  | **Female attention time difference : Courtship duration difference** |  |  |  |  |  | 1.074 |
| **m7** | **Female CW difference +** | 5 | -113.72 | 241.2 | 5.43 | 0.034 | 1.036 |
|  | **Female attention time difference +** |  |  |  |  |  | 1.431 |
|  | **Courtship duration difference** |  |  |  |  |  | 1.419 |
| **m10** | **Female CW difference +** | 5 | -113.72 | 241.2 | 5.44 | 0.034 | 1.027 |
|  | **Courtship duration difference +** |  |  |  |  |  | 1.092 |
|  | **Female attention time difference : Courtship duration difference** |  |  |  |  |  | 1.079 |
| **m5** | **Female attention time difference** | 3 | -125.54 | 258.4 | 22.65 | 0.000 |  |
| **m1** | **Female CW difference + Female attention time difference** | 4 | -125.20 | 260.8 | 25.00 | 0.000 |  |
| **m13** | **Female attention time difference +** | 4 | -125.34 | 261.0 | 25.27 | 0.000 | 1.842 |
|  | **Female attention time difference : Courtship duration difference** |  |  |  |  |  | 1.842 |
| **m0** | **Null model ~ 1** | 2 | -128.43 | 261.5 | 25.72 | 0.000 |  |
| **m8** | **Female attention time difference : Courtship duration difference** | 3 | -127.78 | 262.9 | 27.13 | 0.000 |  |
| **m4** | **Female CW difference** | 3 | -127.80 | 262.9 | 27.18 | 0.000 |  |
| **m9** | **Female CW difference +** | 5 | -125.01 | 263.8 | 28.01 | 0.000 | 1.032 |
|  | **Female attention time difference +** |  |  |  |  |  | 1.881 |
|  | **Female attention time difference : Courtship duration difference** |  |  |  |  |  | 1.843 |
| **m12** | **Female CW difference + Female attention time difference : Courtship duration difference** | 4 | -127.25 | 264.9 | 29.09 | 0.000 |  |
|  |  | | | | | |  |
|  | ***Ph. bifurcilinea*** | | | | | |  |
| **m3** | **Female attention time difference + Courtship duration difference** | 4 | -109.52 | 229.5 | 0.00 | 0.262 |  |
| **m6** | **Courtship duration difference** | 3 | -111.06 | 229.5 | 0.00 | 0.262 |  |
| **m11** | **Female attention time difference +** | 5 | -108.73 | 231.5 | 1.92 | 0.100 | 1.360 |
|  | **Courtship duration difference +** |  |  |  |  |  | 1.194 |
|  | **Female attention time difference : Courtship duration difference** |  |  |  |  |  | 1.206 |
| **m7** | **Female CW difference +** | 5 | -108.75 | 231.5 | 1.97 | 0.098 | 1.068 |
|  | **Female attention time difference +** |  |  |  |  |  | 1.236 |
|  | **Courtship duration difference** |  |  |  |  |  | 1.251 |
| **m15** | **Female CW difference +** | 6 | -106.97 | 231.9 | 2.41 | 0.079 | 1.205 |
|  | **Female attention time difference +** |  |  |  |  |  | 1.507 |
|  | **Courtship duration difference +** |  |  |  |  |  | 1.251 |
|  | **Female attention time difference : Courtship duration difference** |  |  |  |  |  | 1.361 |
| **m2** | **Female CW difference + Courtship duration difference** | 4 | -110.76 | 232.0 | 2.49 | 0.076 |  |
| **m14** | **Courtship duration difference +** | 4 | -110.95 | 232.4 | 2.87 | 0.062 | 1.053 |
|  | **Female attention time difference : Courtship duration difference** |  |  |  |  |  | 1.053 |
| **m10** | **Female CW difference +** | 5 | -110.53 | 235.1 | 5.52 | 0.017 | 1.088 |
|  | **Courtship duration difference +** |  |  |  |  |  | 1.065 |
|  | **Female attention time difference : Courtship duration difference** |  |  |  |  |  | 1.116 |
| **m5** | **Female attention time difference** | 3 | -113.88 | 235.2 | 5.65 | 0.016 |  |
| **m1** | **Female CW difference + Female attention time difference** | 4 | -112.58 | 235.7 | 6.14 | 0.012 |  |
| **m9** | **Female CW difference +** | 5 | -111.36 | 236.7 | 7.20 | 0.007 | 1.150 |
|  | **Female attention time difference +** |  |  |  |  |  | 1.282 |
|  | **Female attention time difference : Courtship duration difference** |  |  |  |  |  | 1.361 |
| **m13** | **Female attention time difference +** | 4 | -113.56 | 237.6 | 8.08 | 0.005 | 1.199 |
|  | **Female attention time difference : Courtship duration difference** |  |  |  |  |  | 1.199 |
| **m0** | **Null model ~ 1** | 2 | -117.16 | 239.0 | 9.45 | 0.002 |  |
| **m4** | **Female CW difference** | 3 | -116.59 | 240.6 | 11.06 | 0.001 |  |
| **m8** | **Female attention time difference : Courtship duration difference** | 3 | -117.10 | 241.6 | 12.08 | 0.001 |  |
| **m12** | **Female CW difference + Female attention time difference : Courtship duration difference** | 4 | -116.59 | 243.7 | 14.15 | 0.000 |  |

CW: carapace width.

**Table S2** Experiment 2: Comparisons of six generalized linear models to predict prey-choice by *Po. xishan*

| **Model** | **Predictor** | **df** | **logLik** | **AICc** | **Delta** | **Weight** | **VIF** |
| --- | --- | --- | --- | --- | --- | --- | --- |
|  | ***Ph. vittata*** | | | | | |  |
| **m4** | **Female movement time : Prey-predator CW ratio** | 2 | -12.63 | 29.7 | 0.00 | 0.486 |  |
| **m0** | **Null model ~ 1** | 1 | -14.55 | 31.3 | 1.53 | 0.227 |  |
| **m2** | **Female movement time** | 2 | -13.94 | 32.4 | 2.62 | 0.131 |  |
| **m1** | **Prey-predator CW ratio** | 2 | -14.52 | 33.5 | 3.78 | 0.073 |  |
| **m5** | **Female movement time +** | 4 | -12.39 | 34.5 | 4.79 | 0.044 | 1.529 |
|  | **Prey-predator CW ratio +** |  |  |  |  |  | 1.021 |
|  | **Female movement time : Prey-predator CW ratio** |  |  |  |  |  | 1.528 |
| **m3** | **Female movement time +** | 3 | -13.93 | 34.9 | 5.14 | 0.037 | 1.022 |
|  | **Prey-predator CW ratio** |  |  |  |  |  | 1.022 |
|  |  | | | | | |  |
|  | ***Ph. bifurcilinea*** | | | | | |  |
| **m4** | **Female movement time : Prey-predator CW ratio** | 2 | -11.92 | 28.3 | 0.00 | 0.671 |  |
| **m2** | **Female movement time** | 2 | -14.13 | 32.7 | 4.41 | 0.074 |  |
| **m0** | **Null model ~ 1** | 1 | -15.75 | 33.6 | 5.32 | 0.047 |  |
| **m3** | **Female movement time +** | 3 | -13.56 | 34.1 | 5.80 | 0.037 | 1.01 |
|  | **Prey-predator CW ratio** |  |  |  |  |  | 1.01 |
| **m1** | **Prey-predator CW ratio** | 2 | -15.35 | 35.2 | 6.85 | 0.022 |  |

CW: carapace width.
